# *Pseudomonas putida* and *Pseudomonas fluorescens* Species Group Recovery from Human Homes Varies Seasonally and by Environment

**DOI:** 10.1371/journal.pone.0127704

**Published:** 2015-05-29

**Authors:** Susanna K. Remold, Megan E. Purdy-Gibson, Michael T. France, Thomas C. Hundley

**Affiliations:** University of Louisville Department of Biology, Louisville, Kentucky, United States of America; Agricultural University of Athens, GREECE

## Abstract

By shedding light on variation in time as well as in space, long-term biogeographic studies can help us define organisms’ distribution patterns and understand their underlying drivers. Here we examine distributions of *Pseudomonas* in and around 15 human homes, focusing on the *P*. *putida* and *P*. *fluorescens* species groups. We describe recovery from 10,941 samples collected during up to 8 visits per home, occurring on average 2.6 times per year. We collected a mean of 141 samples per visit, from sites in most rooms of the house, from the surrounding yards, and from human and pet occupants. We recovered *Pseudomonas* in 9.7% of samples, with the majority of isolates being from the *P*. *putida* and *P*. *fluorescens* species groups (approximately 62% and 23% of *Pseudomonas* samples recovered respectively). Although representatives of both groups were recovered from every season, every house, and every type of environment sampled, recovery was highly variable across houses and samplings. Whereas recovery of *P*. *putida* group was higher in summer and fall than in winter and spring, *P*. *fluorescens* group isolates were most often recovered in spring. *P*. *putida* group recovery from soils was substantially higher than its recovery from all other environment types, while higher *P*. *fluorescens* group recovery from soils than from other sites was much less pronounced. Both species groups were recovered from skin and upper respiratory tract samples from healthy humans and pets, although this occurred infrequently. This study indicates that even species that are able to survive under a broad range of conditions can be rare and variable in their distributions in space and in time. For such groups, determining patterns and causes of stochastic and seasonal variability may be more important for understanding the processes driving their biogeography than the identity of the types of environments in which they can be found.

## Introduction

The distributions of many organisms vary in time as well as space. In microbial ecology and biogeography, studies that include the longitudinal sampling required to address temporal variation have found significant seasonal variation in communities of soils, [1, 2], rhizospheres [3], lakes [4] oceans [5], and even in the indoor built environment [6–9]. A better understanding of how an organism’s use of its environment varies across seasons has important implications for understanding the parameters that are most important in the delineation of its habitat and also of the types and patterns of selection pressures it experiences. In addition, the degree to which environmental variability exists in space vs. in time has been predicted to affect the life history strategies that are most favorable [10, 11].

We focus here on two large species groups within the genus *Pseudomonas*, the *P*. *putida* and *P*. *fluorescens* groups [12]. Representatives from these groups have been isolated from a variety of habitats such as: soils and rhizospheres [13–15], drains[14], fresh and salt water [16–20], plants [21, 22], and in clinical settings [23–25]. Nevertheless, despite substantial overlap in the environments from which they have been isolated, they differ in the resources they are able to use [26, 27], in their temperature tolerances [26, 28], and they have been found to differ in their relative abundances in some types of environments. For example, in a previous study in which we used a similar sampling approach to that described here, we found *P*. *putida* group strains to be more common than *P*. *fluorescens* group strains in household sites [14]; Igbinosa et al [20] found more *P*. *putida* than *P*. *fluorescens* in their freshwater and wastewater samplings; and Negi et al [15] isolated substantially more *P*. *fluorescens* than *P*. *putida* in their collection of cold tolerant Himalayan rhizosphere *Pseudomonas* strains.

In this study we used culture-based methods to characterize the distributions of members of these two species groups in a human household setting. Recovery of *P*. *aeruginosa* from this sampling effort is described elsewhere [29], and, consistent with other studies of human households, indicated that this species differs from other *Pseudomonas* in being almost a drain specialist in a household context [14, 29–32]. We explored a broad range of types of environments both inside and outside the home, in a longitudinal study including 15 households sampled up to 8 times each, over a 4.5-year period. This allowed us to characterize and compare seasonal variation in recovery in these two species groups in a range of types of environments. Even in the indoor built environment, recovery of some microbes has been found to vary seasonally, and is sometimes correlated with outdoor seasonal variability in distributions [6–9]. Inclusion of both indoor and outdoor sampling in this study allowed us to address the possibility that outdoor seasonality may be influencing indoor populations in these two groups of *Pseudomonas*.

## Materials and Methods

### Household Sampling

**Fig 1:**
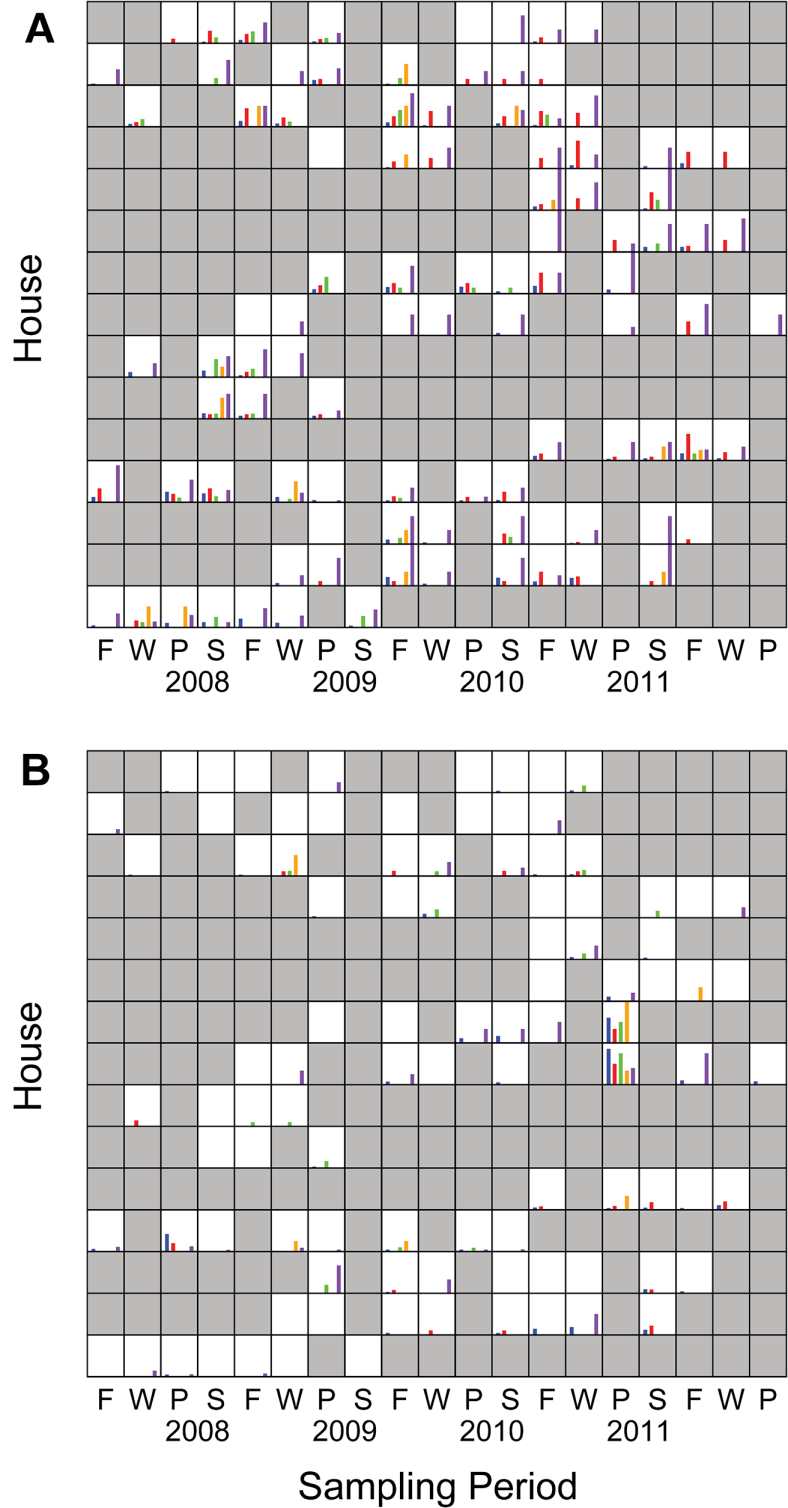
Proportion of sites from which (A) *P*. *putida* group and (B) *P*. *fluorescens* group were recovered by house and sampling period. First sampling season was Fall 2007. Frequently wet sites are shown in blue, drains in red, water in green, garbage and compost in orange, soils in purple. Human and animal sites are not shown. Fields shaded gray indicate that no sampling occurred.

We collected samples from 15 households in the Louisville, KY (USA) metropolitan area. One house in this study was included in a previous study [14], but the sampling results reported for that house in the previous study was not included in this analysis. Houses were each sampled between 3 and 8 times between October 2007 and March 2012, with over half (n = 8) being sampled 8 times (Fig 1). The interval between samplings ranged from 3.5 to 6.1 months, with a mean of 4.6 months. A total of 11,674 samples were taken, but as described below, 733 were eliminated from the analyses presented here resulting in a final data set of 10,941 samples. The raw data describing the characteristics of the sampling time and location, and whether or not each of the focal *Pseudomonas*, *P*. *putida* group and *P*. *fluorescens* group, were recovered was deposited in tab delimited text format in the Dryad Digital Repository (DRYAD), doi:10.5061/dryad.2s361.

Seven households included one child with cystic fibrosis (CF) and 8 had no CF patients. All households with a CF patient were recruited through the Pediatric CF Center at the University of Louisville; households without CF patients were recruited with flyers distributed on the University of Louisville Campus and by word of mouth. Because preliminary analyses of recovery of both *P*. *putida* group and *P*. *fluorescens* group recovery did not differ between these two types of houses (analyses not shown), the two types of houses were combined for the analyses presented here, and sites that are specific to homes with people with CF (upper respiratory samples from CF patients and samples from equipment related to treatment of CF) were removed from the dataset.

At each visit to a house, sampling similar to the method reported in Remold et al. [14]. Within each household, between 74 and 168 samples (mean of 141) were collected, depending on the number of bathrooms, people, pets, etc., from 123 types of sites in and around the home (S1 Table). Subjects enrolled in the study were instructed not to clean the home the week before the sampling date. To minimize the risk of cross-contamination, no two households were sampled on the same day.

### Ethics Statement

The study protocol was approved by the University of Louisville Biomedical Institutional Review Board (IRB #408.06). Written informed consent was obtained from all adult subjects and from a parent of all minors, and written assent was obtained from minors able to provide it. Sampling of animals was approved by the University of Louisville Institutional Animal Care and Use Committee (IACUC #10093).

### Collection Method

Samples were collected with sterile swabs pre-moistened with phosphate-buffered saline. Surfaces were sampled at locations most likely to have frequent contact with human skin (i.e., knobs, buttons, etc.). Soils were sampled by inserting a swab 1–2 inches from the surface, collecting soil on the swab. All drains were swabbed within the first 1–2 inches from the top of the opening, and running water sample were taken by wetting swabs immediately after opening the tap. Some of the subjects or their parents/guardians also collected human fecal and genital samples; these were excluded from all analyses presented here due to low sample size and low recovery rates of *P*. *putida* and *P*. *fluorescens* group isolates.

All swabs were streaked onto *Pseudomonas* isolation agar (PIA, 10 g gelatin peptone, 10 g meat peptone, 10 g potassium sulfate, 1.4 g magnesium chloride, 25 mg Irgasan, 20 ml glycerol, 13.6 g agar, 1 L water; based on [33]) at the homes. PIA was used in this study because the large number of samples taken and the expected relatively low rate of recovery from some of our environments necessitated a selection that could be used to allow the growth of most *Pseudomonas* while eliminating most *Pseudomonas*-negative swabs without molecular identification. We note however that PIA has limitations with respect to both these objectives Ghyselinck et al [34], have found that the diversity of *Pseudomonas* that grow on PIA is lower than that obtained on the non-selective media TSA and PDA, and many non-*Pseudomonas* were obtained from our PIA plates.

Plates were transported on ice and incubated for 48 hours at 28°C immediately upon return to the laboratory. Plates without growth were incubated for an additional 24 hours before being scored as *Pseudomonas* negative. We chose incubation at 28°C for three reasons. First, other studies suggest that a number of *Pseudomonas* have wide ranges of temperature tolerance, with 25–30°C permitting growth of all studied strains [26, 35]. Second, preliminary experiments in which we isolated strains from household sites at both 28°C and 37°C yielded more strains at the lower temperature, and whereas all strains isolated at 37°C also grew at 28°C, the reverse was not true. Others have also shown that Pseudomonas isolated from cold sites (0–15°C) can be recovered at 25°C [36]. Finally, among our non-host associated sites, all but a small minority (those associated with refrigerators and some basements and cellars) either spend most of the year at temperatures between 25 and 30°C (most indoor samples), or experience temperatures in this range regularly for at least part of the year (outdoor samples)

Where growth occurred, a single colony from each plate was picked randomly, re-streaked onto PIA. Following isolation on PIA and successful growth when re-streaked on PIA, some non-*Pseudomonas* were excluded using a MacConkey screen for lactose fermentation (MacConkey agar: 17 g pancreatic digest of gelatin, 1.5 g pancreatic digest of casein,1.5 g peptic digest of animal tissue,10 g lactose,1.5 g bile salts mixture, 5 g sodium chloride, 0.03 g neutral red, 0.001 g crystal violet, 13.5 g agar, 1 L water, based on [33]). We also employed a PCR-based screen using *Pseudomonas*-specific primers [37]. Where multiple colony morphologies grew from a single sample, one of each was frozen for further analysis. From among these cases there were 22 instances in which a member of two different species groups were identified from the same sample. Of these, 12 are cases in which a *P*. *putida* group and a *P*. *fluorescens* group isolate were obtained from the same sample.

### Sample Identification

Strains identified as candidate *Pseudomonas* using the selections and screens described above were identified using a combination of *Pseudomonas* specific primers [37] and 16S rDNA sequencing (either on its own, or to follow up on isolates identified as *Pseudomonas* using genus-specific primers). For sequence-based identification, fragments amplified with universal bacterial primers (8f and 1492r) were sequenced with a 1401r primer or 8f primer [38]. Sequences at least 500bp in length were then compared to the database Bioinfo 1200 nucleotide [39]. Identifications were then made a second time with the database EzTaxon [40] to determine consistency across databases. The species identifications made with the Bioinfo 1200 nucleotide database were used only as a step in assigning each strain to a species group as defined by Anzai et al [12]. Isolates that were identified by comparison to this database as members of species not classified by Anzai et al [12] were assigned to a species group by generating a provisional assignment of the unclassified species as follows: the 16S type sequence of the species to which the isolate was assigned was blasted against the NCBI database. The unclassified species was then assigned to the species group of the most similar Anzai et al. [12]-classified species among the top blast hits. We obtained four strains with best-match species that Anzai et al [12] did not group, and there were two sequences of insufficient quality to assign to a group (the latter were treated as missing data with respect to *P*. *putia* and *P*. *fluorescens* group recovery). The partial 16S rDNA sequence used to identify all *Pseudomonas* obtained from the samples included in this study are available from GenBank under accession numbers KP452510—KP453692.

Assignment of all 251 strains to the *P*. *fluorescens* group was consistent between the two databases, Bioinfo 1200 nucleotides and EzTaxon. However, among the 668 strains classified in *P*. *putida* group there were 33, collected from a range of houses and environment types, that were all identified by Bioinfo 1200 as *P*. *fulva*, a species in the *P*. *putida* group, but by EzTaxon as members a number of different species that we had assigned to *P*. *aeruginosa* group through the method described above. In many cases, the 10 most similar sequences identified by both Bioinfo 1200 and EzTaxon included members of both *P*. *putida* and *P*. *aeruginosa* groups. We there fore performed all statistical analyses twice, with and without including these 33 strains among the *P*. *putida* group isolates. Because few qualitative differences emerged between these parallel analyses, only those conducted on the dataset including the 33 strains within *P*. *putida* group are presented here.

### Statistical Analysis

For analyses and for graphing we created the predictor variable “environment type” by binning the 123 types of sites sampled (S1 Table). The variable “sampling period” was created by binning sampling dates into season-year combinations, (e.g. Fall 2007), with which seasons defined as follows: winter, December-February; spring, March-May; summer, June-August; fall, September-November. A single sampling intended to occur in the fall but which had to be rescheduled to December 7 was coded as Fall for analysis to distinguish it from the sampling of the same house that occurred later that winter.

In parallel models, we fitted presence or absence of *P*. *putida* group or *P*. *fluorescens* group as the binary response variable, environment type and sampling period as fixed predictor variables, house and its interaction with environment and sampling period as random factors, and we accommodated repeated samplings of individual sampled sites at different sampling periods using an autoregressive order 1 (AR(1)) R-side covariance structure, using a general linear mixed model (PROC GLIMMIX, SAS / STAT 9.3 [41]). An analogous model was used to explore differences in relative recovery between the two species groups, *P*. *putida* and *P*. *fluorescens*. In this model we considered those sites from which either *P*. *putida* group or *P*. *fluorescens* group was recovered, and looked at the probability of recovering one vs. the other. For this analysis we therefore excluded all sites from which both isolates were found, those from which only members of other species groups were found, and those from which no *Pseudomonas* were found. We ran separate models using the data set as a whole, and using the outdoor and indoor subsets of the data set. Additional sub-models describe only spring samplings, and only fall samplings. Some random interaction terms were excluded from some sub-models when their inclusion caused models to fail to converge. Also, because categories for which no recoveries occur cause these models to fail to converge, some houses and/or sampling periods were excluded from some models.

Hypotheses regarding seasonal differences were tested using linear combinations of least squares means of sampling periods. The interaction between sampling period and environment type was not tested because there were many combinations for which no *P*. *putida* group and/or *P*. *fluorescens* group was obtained, and this situation causes the models used here to fail to converge. Random factors were tested using Wald Z tests, and least squares means estimates were used to generate estimated probabilities of recovery, odds of recovery, and odds ratios.

## Results

### Overview of the strain collection

*Pseudomonas* species were recovered from every season, every house, and every environment type sampled (S1 Table). Out of 10,941 samples, 1,056 (9.7%) yielded *Pseudomonas* isolates. Twenty-two samples from which more than one morphologically distinguishable colony types were collected yielded isolates from two different species groups, resulting in a total of 1,078 clearly distinct *Pseudomonas* isolates in our collection. Of these 62% were from the *P*. *putida* group and 23% were from the *P*. *fluorescens* group. Recovery from the next most common species group, *P*. *aeruginosa* group, made up only 13% of isolates, and the remaining 2% of isolates (15 in total) included representatives from a number of species groups as well as some strains not assigned to any species group. Patterns of recovery of *P*. *putida* and *P*. *fluorescens* groups are highly variable across houses and sampling periods

Recovery of *P*. *putida* group or *P*. *fluorescens* group in consecutive samplings was significantly correlated in all but one model evaluated (Fig 1, AR(1) covariance terms in Tables 1–3). Nevertheless, there was also significant stochasticity in the distribution of both groups. Notably, most models detected strong sampling period-specific and/or environment type-specific among-house variation in recovery (Tables 1–4). What follows describes the additional variation attributable to the fixed predictor variables season and environment type, which exists despite this very strong variability in recovery.

**Table 1:** General linear mixed models testing fixed and random predictors of recovery of *P*. *putida* group strains, *P*. *fluorescens* group strains, and their relative recovery, across samples taken from all homes and all sites within homes, in all sampling periods.

	*P*. *putida* group^a^		*P*. *fluorescens* group^b^		Relative recoveries^c^	
Source	DFǂ	Test Statistic§	DFǂ	Test Statistic§	DFǂ	Test Statistic§
Sampling period	18, 10579	3.94***	17, 10457	2.39**	17, 851	3.66***
Season	3, 10579	13.25***	3, 10457	2.81*	3, 851	9.93***
Environment	9, 10579	39.85***	9, 10457	6.40***	9, 851	4.38***
House		1.57+		1.20NS		1.45+
House*Sampling period		2.21*		3.70***		1.46+
House*Environment		3.27***		4.14***		1.45+
AR(1) Covariance Structure, repeated measures on sites		5.40***		4.00***		0.36NS

^a^ models recovery of *P*. *putida* group among all samples.

^b^ models recovery of *P*. *fluorescens* group. One summer sampling excluded due to absence of any *P*. *fluorescens* group recovery.

^c^ models recovery of *P*. *putida* group vs. *P*. *fluorescens* group, given that one of the two was recovered. One summer sampling excluded due to absence of any *P*. *fluorescens* group recovery.

ǂ DF indicates degrees of freedom, denominator DF for F test is estimated using the Residual DF method.

§ The fixed effects are tested with an approximate F test. The random effects are tested using Wald Z tests.

# The effect of season is tested using a joint test for the six pairwise comparisons among seasons, using linear combinations of the least squares means.

NS p>0.1;

+ 0.05<p<0.1;

* 0.01<p<0.05;

** 0.001<p<0.01;

*** p<0.001

**Table 2:** General linear mixed models testing fixed and random predictors of recovery of *P*. *putida* group strains, *P*. *fluorescens* group strains, and their relative recovery, across samples taken from outdoor sites from all homes.

	*P*. *putida* group^a^		*P*. *fluorescens* group^b^		Relative recoveries^c^	
Source	DFǂ	Test Statistic§	DFǂ	Test Statistic§	DFǂ	Test Statistic§
Sampling period	18, 778	2.48***	15, 663	1.08NS	15, 168	1.85*
Season	3, 778	8.00***	3, 663	1.00NS	3, 168	4.06**
Environment	4, 778	20.79***	4, 663	1.84NS	4, 168	2.34+
House		-0.39NS		0.62NS		-1.55NS
House*Sampling period		2.27*		na		na
House*Environment		1.19NS		na		na
AR(1) Covariance Structure, repeated measures on sites		4.90***		0.02NS		10.4***

^a^ models recovery of *P*. *putida* group; includes all samplings of all houses.

^b^ models recovery of *P*. *fluorescens* group. Interactions involving house excluded due to failure of model convergence. One house, two summer sampling periods and one spring sampling period excluded due to absence of any outdoor *P*. *fluorescens* group recovery.

^c^ models recovery of *P*. *putida* group vs. *P*. *fluorescens* group, given that one of the two was recovered. Two houses, one spring, one summer and one fall sampling period excluded due to absence of any samples from which a *P*. *putida* or *P*. *fluorescens* group isolate was recovered.

ǂ DF indicates degrees of freedom, denominator DF for F test is estimated using the Residual DF method.

§ The fixed effects are tested with an approximate F test. The random effects are tested using Wald Z tests.

# The effect of season is tested using a joint test for the six pairwise comparisons among seasons, using linear combinations of the least squares means

NS p>0.1

+ 0.05<p<0.1

* 0.01<p<0.05

** 0.001<p<0.01

*** p<0.001

**Table 3:** General linear mixed models testing fixed and random predictors of recovery of *P*. *putida* group strains, *P*. *fluorescens* group strains, and their relative recovery, across samples taken from indoor, human, and animal sites from all homes.

	*P*. *putida* group^a^		*P*. *fluorescens* group^b^		Relative recoveries^c^	
Source	DFǂ	Test Statistic§	DFǂ	Test Statistic§	DFǂ	Test Statistic§
Sampling period	17, 9700	2.53***	17, 8642	2.14**	16, 598	2.18**
Season	3, 9700	8.94***	3, 8642	1.70NS	3, 598	4.24**
Environment	9, 9700	20.73***	9, 8642	3.90***	9, 598	3.38***
House		1.40NS		1.19NS		1.13NS
House*Sampling period		2.37*		3.51***		2.29*
House*Environment		3.16**		3.57***		1.85+
AR(1) Covariance Structure, repeated measures on sites		4.67***		4.65***		-0.08NS

^a^ models recovery of *P*. *putida* group; includes all houses and sampling periods.

^b^ models recovery of *P*. *fluorescens* group. Two houses and one summer sampling period excluded due to absence of any indoor, human or animal *P*. *fluorescens* recovery.

^c^ models recovery of *P*. *putida* group vs. *P*. *fluorescens* group, given that one of the two was recovered. Two houses, one spring and one summer sampling period excluded due to absence of any samples from which a *P*. *putida* or *P*. *fluorescens* group isolate was recovered indoors or from humans or animals.

ǂ DF indicates degrees of freedom; denominator DF for F test is estimated using the Residual DF method.

§ The fixed effects are tested with an approximate F test. The random effects are tested using Wald Z tests.

# The effect of season is tested using a joint test for the six pairwise comparisons among seasons, using linear combinations of the least squares means

NS p>0.1

+ 0.05<p<0.1

* 0.01<p<0.05

** 0.001<p<0.01

*** p<0.001

**Table 4:** General linear mixed models testing fixed and random predictors of recovery of *P*. *putida* group strains, *P*. *fluorescens* group strains, and their relative recovery, in spring (top) and fall (bottom) sampling periods.

	*P*. *putida* group^a^		*P*. *fluorescens* group^b^		Relative recoveries^c^	
Source	DFǂ	Test Statistic§	DFǂ	Test Statistic§	DFǂ	Test Statistic§
***Spring recovery***						
Sampling period	4, 1237	0.81NS	4, 1113	3.04*	4, 172	5.30***
Environment	5, 1237	16.03***	5, 1113	1.01NS	5, 172	3.57**
House		0.89NS		0.27NS		1.47NS
House*Sampling period		1.07NS		0.94NS		na
House*Environment		-0.07NS		1.10NS		0.46NS
AR(1) Covariance Structure, repeated measures on sites		0.48NS		0.14NS		-0.63NS
		0.48NS		0.14NS		-0.63NS
***Fall recovery***						
Sampling period	4, 2068	3.74**	4, 1791	1.96+	4, 269	17.09NS
Environment	5, 2068	25.58***	5, 1791	1.06NS	5, 269	2.87*
House		1.64NS		0.66NS		2.06*
House*Sampling period		0.07NS		-0.17NS		0.00 NS
House*Environment		1.34NS		2.34*		-0.07NS
AR(1) Covariance Structure, repeated measures on sites		4.31***		-0.28NS		0.72NS

^a^ models recovery of *P*. *putida* group. One house excluded in spring model.

^b^ models recovery of *P*. *fluorescens* group. Two houses excluded in spring and three houses in fall.

^c^ models recovery of *P*. *putida* group vs. *P*. *fluorescens* group, given that one of the two was recovered, for spring and fall.

na indicates that this term was excluded from the model due to failure to converge of the more complex model

ǂ DF indicates degrees of freedom; denominator DF for F test is estimated using the Residual DF method.

§ The fixed effects are tested with an approximate F test. The random effects are tested using Wald Z tests.

# The effect of season is tested using a joint test for the six pairwise comparisons among seasons, using linear combinations of the least squares means

NS p>0.1

+ 0.05<p<0.1

* 0.01<p<0.05

** 0.001<p<0.01

*** p<0.001

### Seasonal differences in recovery of *P*. *putida* and *P*. *fluorescens* groups

**Fig 2:**
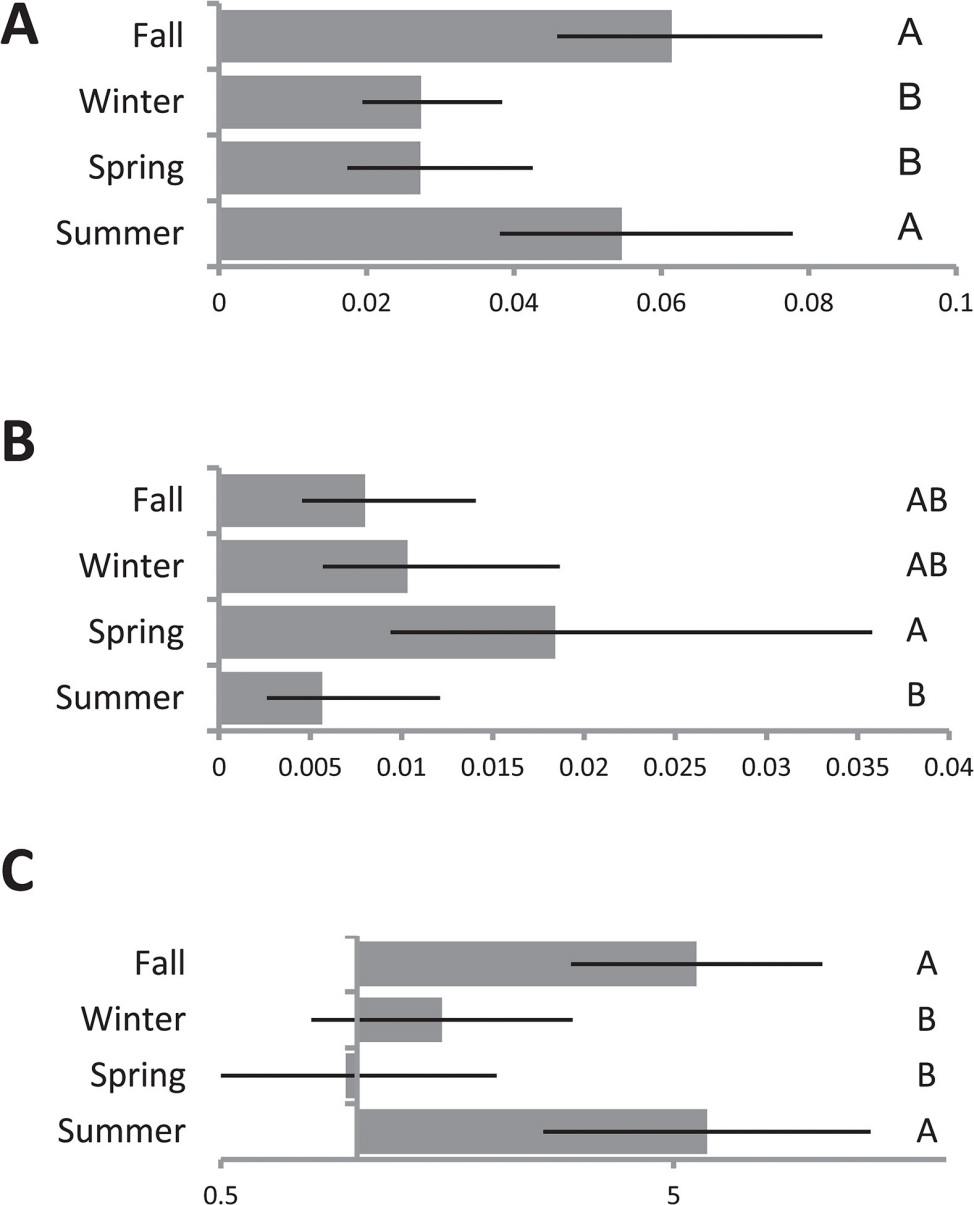
Seasonal differences in recovery. Estimated probability of recovery by season from general linear mixed model describing recovery across all sites, sampling periods and houses (See Table 1), of (**A**) *P*. *putida* group, (**B**) *P*. *fluorescens* group, and (**C**) odds of recovering *P*. *putida* group (as opposed to *P*. *fluorescens* group), from among sites where one or the other was recovered. Error bars indicate 95% confidence intervals. Levels that do not share a letter are significantly different at p<0.05 after Bonferroni adjustment for multiple comparisons.

We used linear combinations of least squares means of sampling periods to generate estimated recovery rates by season and to test for differences among them. Overall, we found that *P*. *putida* group recovery was approximately twice as high in summer and fall than in winter and spring (Table 1, Fig 2A). In contrast, the probability of *P*. *fluorescens* group was significantly more likely to be recovered in the spring than summer, with winter and fall intermediate (Table 1, Fig 2B). Considering those samples from which one of the two was recovered, we found that odds of recovering *P*. *putida* group rather than *P*. *fluorescens* group was 5.5–6 in fall and summer. However, in winter and spring, there was no significant difference in the likelihood of recovering one species group vs. the other (Table 1, Fig 2C).

**Fig 3:**
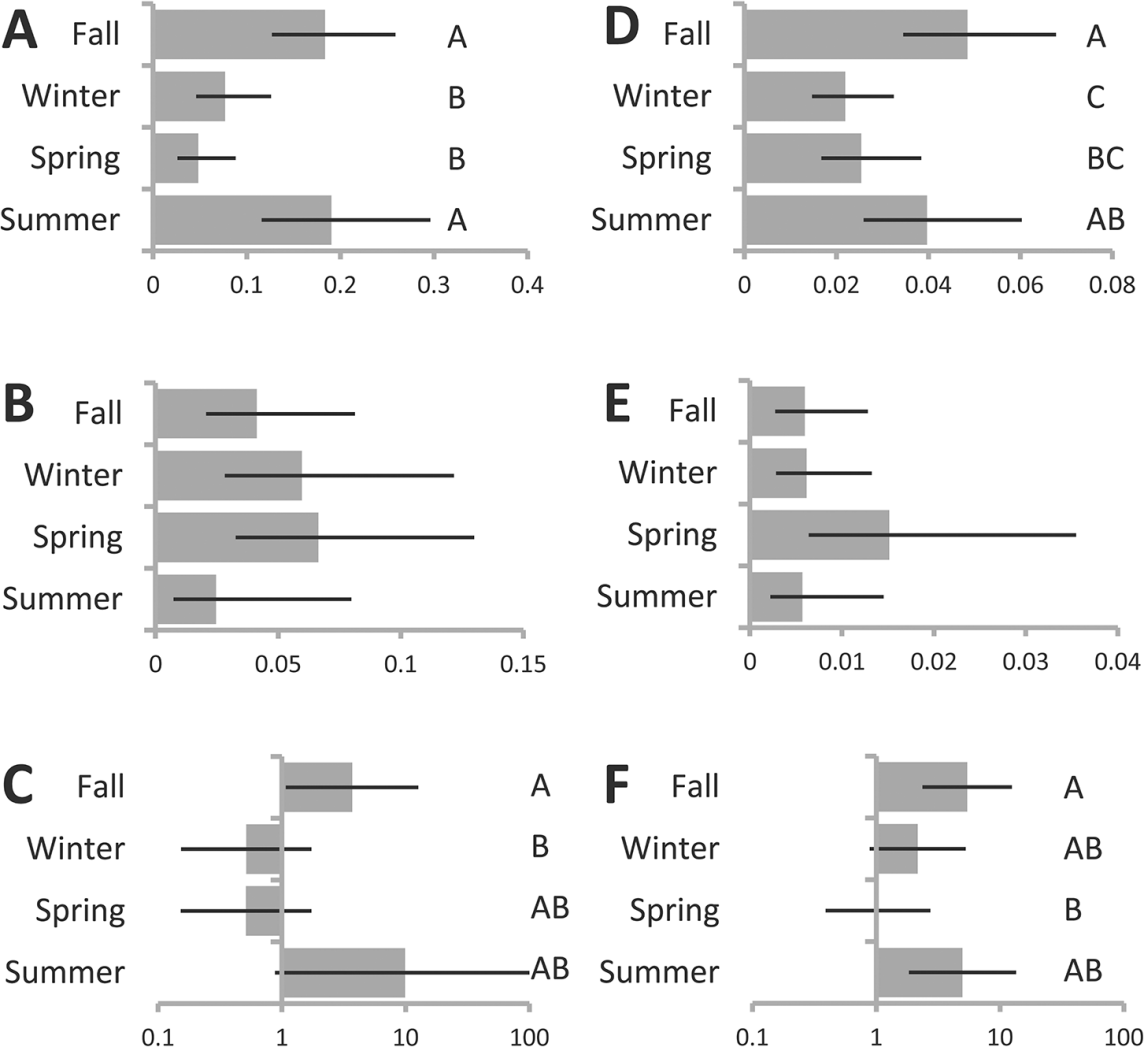
Seasonal differences in recovery in indoor and outdoor subsamples. Estimated probability of recovery by season from general linear mixed model describing recovery across outdoor (**A-C**) and indoor (**D-F**) sites (See Table 2), of (**A, D**) *P*. *putida* group, (**B, E**) *P*. *fluorescens* group strains, and (**C, F**) odds of recovering *P*. *putida* group (as opposed to *P*. *fluorescens* group), from among sites where one or the other was recovered. Levels that do not share a letter are significantly different at p<0.05 after Bonferroni adjustment for multiple comparisons. Error bars indicate 95% confidence intervals. Comparisons in panels without levels indicated yielded no significant differences after correction for multiple comparisons.

We used additional models to explore the degree to which seasonal differences were driven by outdoor samplings. Outdoor recovery of both groups as well as their relative recoveries were consistent with results of analyses of the data overall. *P*. *putida* group recovery from outdoor samples was highest in summer and fall (Table 2, Fig 3A), and although there were no significant differences among seasons in outdoor recovery of *P*. *fluorescens* group the overall trend of higher recovery in spring relative to summer persisted (Table 2, Fig 3B). Among outdoor samples from which one of the two was recovered *P*. *putida* group was more likely to be recovered in summer and fall and *P*. *fluorescens* group was more likely to be recovered in winter and spring, though only difference between fall and winter is statistically significant (Table 2, Fig 3C).

We then considered only indoor samples and samples taken from humans and pets and found that indoor recovery was also consistent with trends in the dataset as a whole. Indoor sites were significantly more likely to yield *P*. *putida* group in fall and summer than in spring, with winter intermediate (Table 3, Fig 3D), and the non-significant trend of highest *P*. *fluorescens* group recovery in spring than in other seasons remains (Table 3, Fig 3E). Trends in relative recovery were also consistent the dataset as a whole in that the dominance of *P*. *putida* group is estimated to be strongest in summer and fall (Table 3, Fig 3F).

### Differences among types of environments in recovery of *P*. *putida* and *P*. *fluorescens* groups

**Fig 4:**
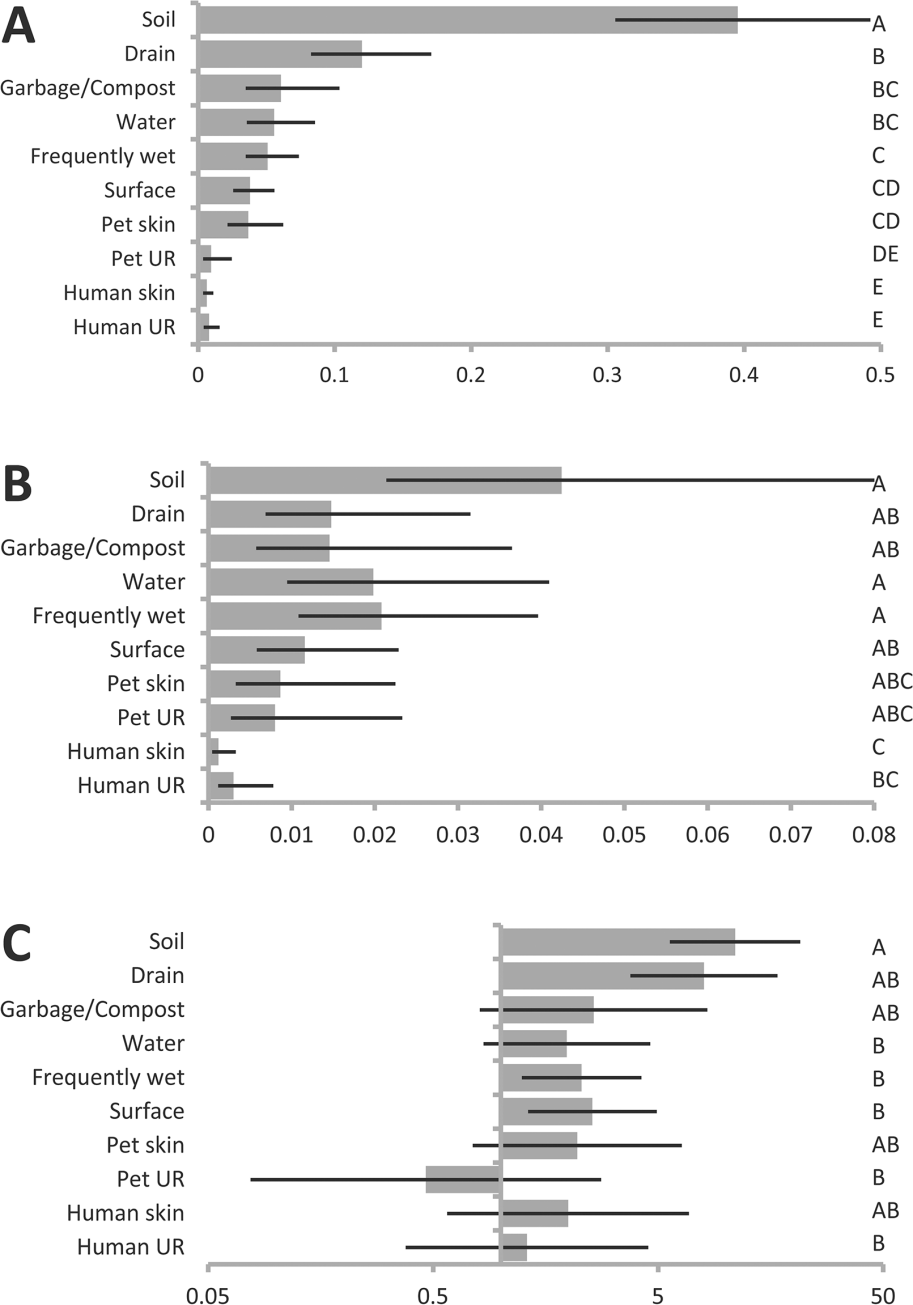
Differences in recovery among environment types. Estimated probability of recovery by environment type from general linear mixed model describing recovery across all sites (See Table 1), of (**A**) *P*. *putida* group, (**B**) *P*. *fluorescens* group strains, and (**C**) odds of recovering *P*. *putida* group (as opposed to *P*. *fluorescens* group), from among sites where one or the other was recovered. Error bars indicate 95% confidence intervals. Levels that do not share a letter are significantly different at p = 0.05 after Bonferroni adjustment for multiple comparisons.

In the data set as a whole, *P*. *putida* group recovery from soils was almost four times higher than recovery from drains, which in turn was also higher than recovery from most other environment types (Table 1, Fig 4). In contrast, *P*. *fluorescens* group recovery was more similar across environment types, though as was the case for *P*. *putida* group, soil recovery was higher than recovery from other environment types (Table 1, Fig 4). Although recovery from human and pet upper respiratory and skin sites was low for both species groups, some *P*. *fluorescens* group and *P*. *putida* group isolates were recovered from all of these four environment types. Considering only samples from which one of the two was recovered, *P*. *putida* group was approximately 8 to 10 times more likely in soil samples than *P*. *fluorescens* group (Table 1, Fig 4), but in a number of other environment types estimated recovery of *P*. *putida* and *P*. *fluorescens* groups relative to the other was not statistically different from equal.

**Fig 5:**
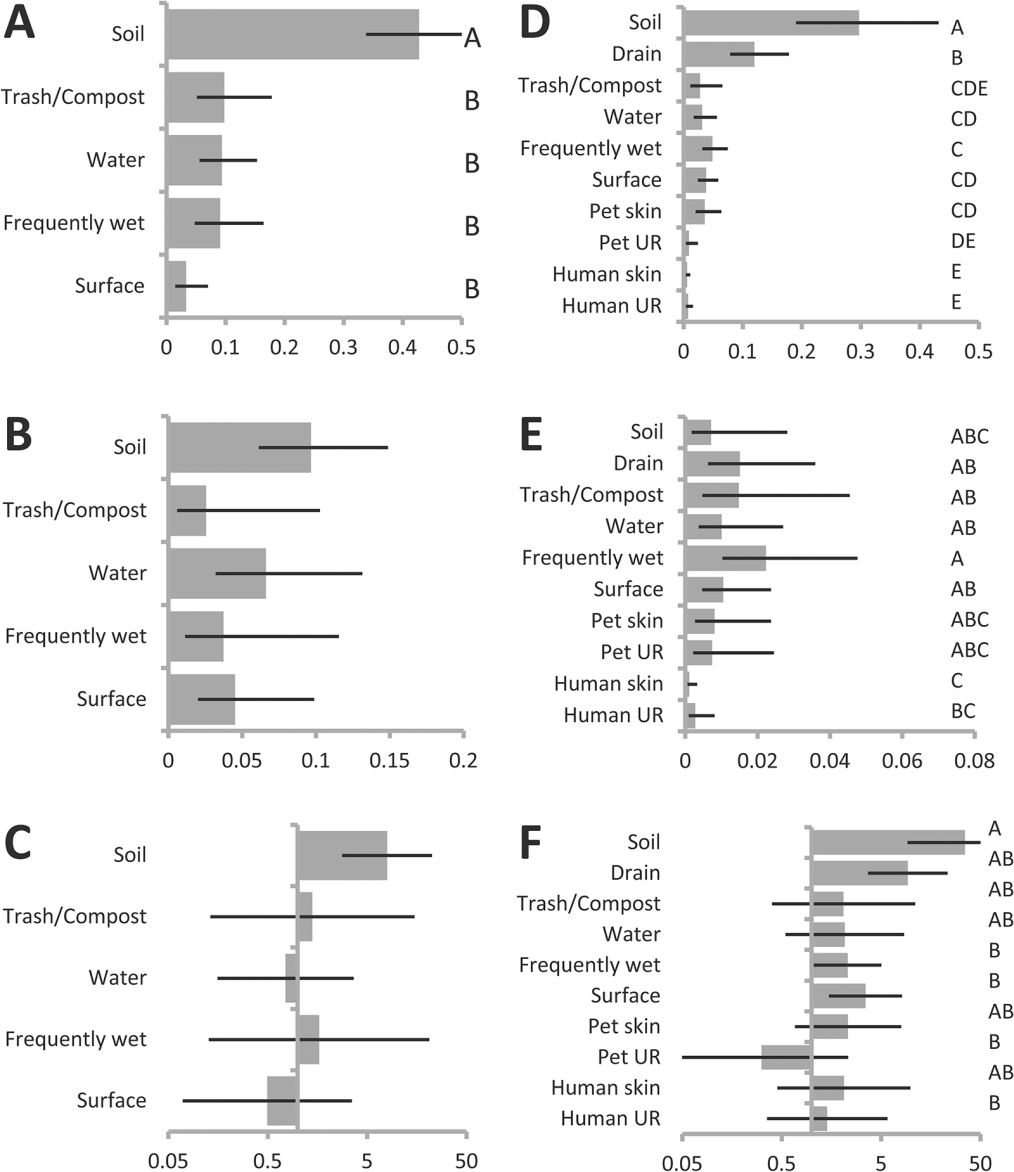
Differences in recovery among environment types in indoor and outdoor subsamples. Estimated probability of recovery by environment type from general linear mixed model describing recovery across outdoor (**A-C**, See Table 2) and indoor (**D-F**, See Table 3) sites, of (**A,D**) *P*. *putida* group, (**B,E**) *P*. *fluorescens* group strains, and (**C,F**) odds of recovering *P*. *putida* group (as opposed to *P*. *fluorescens* group), from among sites where one or the other was recovered. Error bars indicate 95% confidence intervals. Levels that do not share a letter are significantly different at p = 0.05 after Bonferroni adjustment for multiple comparisons. Comparisons in panels without levels indicated yielded no significant differences after correction for multiple comparisons.

The ranking of environments with respect to probability of yielding of *P*. *putida* group was consistent indoors vs. outdoors, though overall recovery for both species groups was higher outdoors (Tables 2 and 3, Fig 5A and 5D). For *P*. *fluorescens* group there were few (indoors, Table 3, Fig 5E), or no (outdoors, Table 2, Fig 5B) significant differences in recovery among environment types. Among outdoor samples there were no significant differences in the odds of recovering one species group vs. the other (Table 2, Fig 5C), but indoors the pattern of variability in relative rates of recovery across environments was highly consistent with the sample overall (Table 3, Fig 5F).

**Fig 6:**
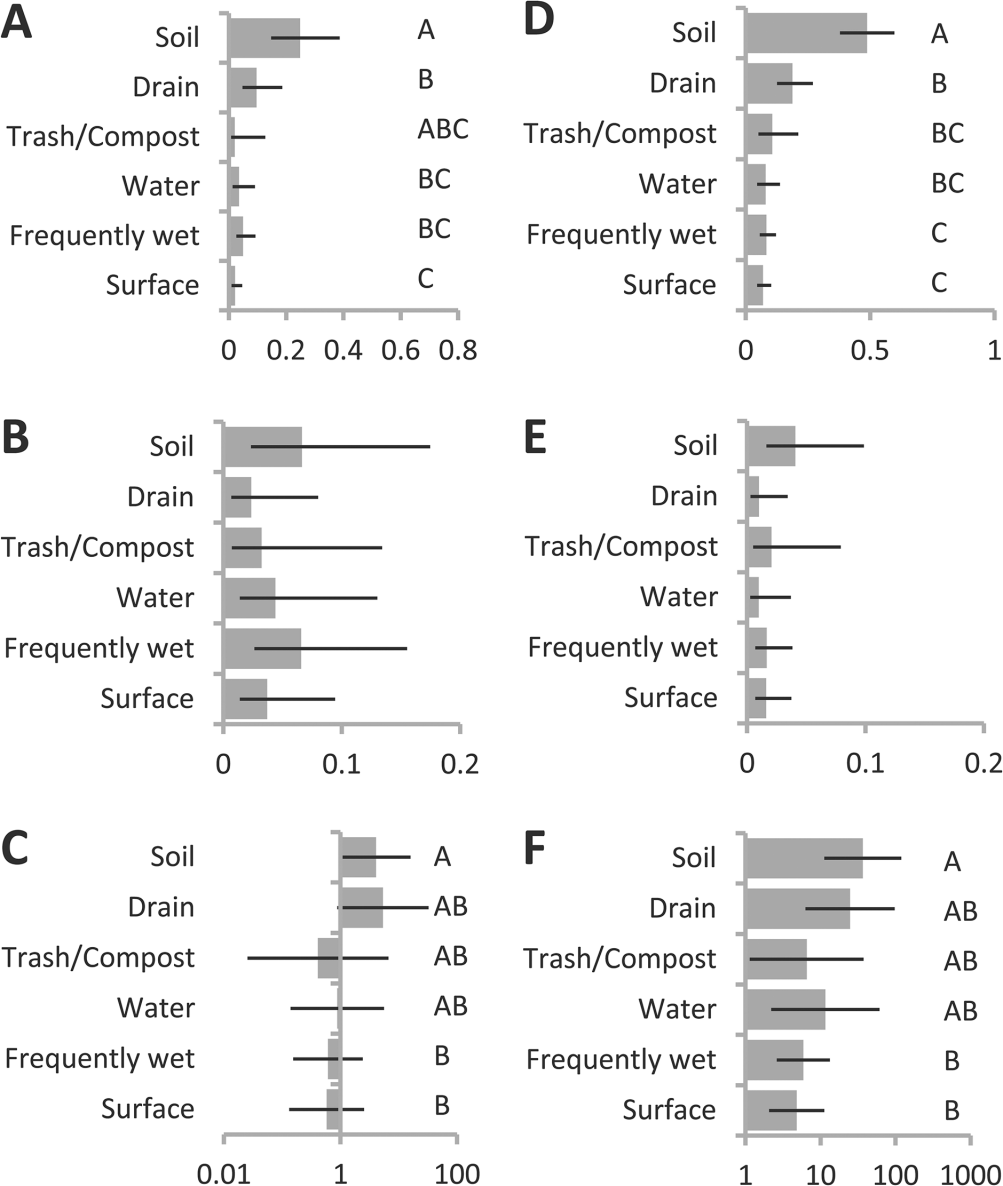
Differences in recovery among environment types in fall and spring subsamples. Estimated probability of recovery by environment type from general linear mixed model describing recovery across sites sampled in spring (**A-C**) and fall (**D-F**), of (**A,D**) *P*. *putida* group, (**B,E**) *P*. *fluorescens* group strains, and (**C,F**) odds of recovering *P*. *putida* group (as opposed to *P*. *fluorescens* group), from among sites where one or the other was recovered. Error bars indicate 95% confidence intervals. Levels that do not share a letter are significantly different at p = 0.05 after Bonferroni adjustment for multiple comparisons. Comparisons in panels without levels indicated yielded no significant differences after correction for multiple comparisons.

It was not possible to test for season-specific differences in recovery among environment types in our full model due to an excess of combinations of these factors from which no isolates were obtained. We therefore ran separate sub-models on samplings occurring in fall and in spring only. These models excluded human and animal-associated samples due to low recovery rates. For *P*. *putida* group, the relative ranking of the environment types was consistent between fall and spring, though estimated recoveries in the spring were on average less than half those of the fall (Fig 6A vs. 6D), and the reverse was true for *P*. *fluorescens* group (Fig 6B vs. 6E). Whereas in the fall, the estimated likelihood of recovering *P*. *putida* group was significantly greater than that of recovering *P*. *fluorescens* groups (with estimates ranging from 4.5 times more likely on surfaces, to over 36 times more likely in soils), in the spring recovery from all habitat types except soil and drains was not different from equal, with estimates suggesting *P*. *fluorescens* may be more common than *P*. *putida* in many of these types of sites (Table 4, Fig 6).

## Discussion

In this study we took 10,941 samples taken from over 100 types of sites in 15 homes, across 8 years, with the goal of investigating the temporal and spatial variation in distribution patterns of *P*. *putida* and *P*. *fluorescens* group *Pseudomonas*. We found striking differences in rates of recovery between *P*. *putida* and *P*. *fluorescens* groups overall, as well as differences in their patterns of seasonal variation, and differential use of environment types. While it is unlikely that artifacts of our culture-based our sampling methods could generate the temporal and spatial patterns observed, two factors result in our absolute recovery rates being biased toward underestimation of the absolute rate of *Pseudomonas* occurrence. First, our culture media, PIA, excludes some *Pseudomonas* [34], and this media and/or our culture temperature may cause underestimation and/or bias in detection of *P*. *putida* and *P*. *fluorescens* groups through exclusion of genotypes. Second, because we identified only one strain per colony morphology on each plate, growth of non-*Pseudomonas* on our PIA plates likely also contributed to underestimation of *Pseudomonas* occurrence.

With this limitation in mind, we note that our finding that *Pseudomonas* were obtained from all environment types sampled, and yet were below the limit of detection in most individual samples suggests they can occur in most sites, but are either rare or undergo frequent extinction and recolonization. This hypothesis is consistent with the results of Dunn et al (2013), who found that the mean relative abundance of all Pseudomonales among high throughput sequence reads obtained from nine household site types was low (5.5–10.8% in kitchen sites, and 1.2–3.3% in other types of sites). Taken together these studies suggest that any one species or species group of *Pseudomonas* may be sufficiently rare that high throughput sequence approach using *Pseudomonas*-specific primers for a region that yields greater taxonomic resolution, such as rpoD, [34] may be necessary to accurately quantify abundance at the species level. This approach will also aid in determining whether populations are small but permanent or go through rounds of extinction and recolonization. Resolving these issues will contribute to uncovering the relative roles of possible drivers of differential recovery discussed below.

The overall difference in relative recovery of *P*. *putida* and *P*. *fluorescens* groups is consistent with previous results [14]. It occurred despite the fact that members of both species groups can use a broad range of resources, and the fact that strains of both have been isolated from many different environments, including from all of the environment types sampled in this study [14]. Therefore, although they both use many types of household sites, in a given sampled area, *P*. *putida* group members are either more abundant or easier to obtain than *P*. *fluorescens* group members. If the former is important, it may reflect difference in the density to which these organisms commonly grow, perhaps as a result in differences in their interactions with the substrate on which they are growing, or with other members of the community. As discussed below, because these two species group differ with respect to which season they are most abundant, studies that sample in just one season may also underestimate *P*. *fluorescens* group use of household habitats.

Our findings also indicate that household sites differ in their suitability for *Pseudomonas*, and that they do so in a taxon-specific way. Members of both species groups were recovered from all environment types, and both were more frequently recovered from soils than from other sites, but whereas soil recovery, followed by drain recovery substantially exceeded recovery in other environment types for *P*. *putida* group, for *P*. *fluorescens* group recovery was much more similar among environment types. The relative rate of recovery of *P*. *putida* group vs. *P*. *fluorescens* group also varied among environment types, with soils being significantly more biased toward *P*. *putida* group than transiently wet sites and surfaces (Tables 1–4, Figs 4–6). *P*. *aeruginosa*, a member of the *P*. *aeruginosa* species group exhibits yet another distinct recovery pattern: in a household setting, it is most commonly found in drains [14, 29–32]. These findings are consistent with that of Remold et al [14], who found that some *Pseudomonas* species were over- or under- represented in some environments relative to their representation across a range of household environments.

There were distinct seasonal trends in the distributions of these two species, with *P*. *putida* group recovery highest in fall and summer, and *P*. *fluorescens* group recovery highest in spring and to a lesser extent in winter, resulting in much greater dominance of *P*. *putida* group over *P*. *fluorescens* group in fall than in spring (Tables 1–4). Higher *P*. *fluorescens* group recovery in spring and winter is consistent with this species being psychrophilic, as a number of strains of this species have been found to be [15, 26, 42, 43]. While the driver of the substantial decrease in *P*. *putida* group recovery in spring relative to fall is unclear, it is consistent with results from South Carolina stream samples, from which more *P*. *putida* recovery was also higher in the fall than in winter or spring [18].

For both species groups, seasonal patterns outdoors and indoors were similar. The finding that sampling inside homes showed seasonal variation (Table 3, Figs 3 and 4) is consistent with the observation of seasonal variation in a number of studies of the built environment [6–9]. Nearby external inputs have been shown to play an important role in shaping the microbial communities found in the built environment, including in homes (e.g. [7, 44]). While it is possible that in the homes we studied the similarity in recovery trends indoors and outdoors is driven by frequent movement of *Pseudomonas* from outside the home to inside, abiotic factors inside the home, or interactions with other organisms that exhibit seasonal variation of movement from outdoors to indoors could also play a role.

In conclusion, we found striking differences between the *P*. *putida* and *P*. *fluorescens* groups in their recovery overall, and across seasons and environment types. This could be driven by a number of factors [45]. First, differences in dispersal opportunities could affect access to household sites. This might, for example, explain higher overall recovery outdoors than indoors. Second, species sorting driven by differences in how members of the two species groups interact with features of the abiotic environment may influence relative recoveries. For example, there may be differences between *P*. *putida* group and *P*. *fluorescens* group in sensitivity to stresses such as extreme temperatures or desiccation. If so, this could generate seasonal differences or differences in relative abundance among sites. In addition, direct competition could drive niche partitioning between members of these species groups. Finally, interactions with other community members, such as phages (both species groups as well as *P*. *aeruginosa* have been shown to be vulnerable to phage [46–48]) or predators such as fungi or amoebae could contribute to the differential use of household environments by these two species groups. Regardless of the underlying mechanism, our study shows that putative generalists with apparently broad habitat use patterns may nevertheless exhibit complex differences in habitat use, and highlights the importance of considering seasonal variation even in studies of indoor environments.

## Supporting Information

S1 Table Sites sampled in the homes by environment type bin. Upper respiratory sites were sampled in people with and without cystic fibrosis (CF); only those from people without CF were included in the analyses presented here. CF equipment sites were obtained from a subset of houses and were also not included in analyses presented here. Fecal and genital samples were obtained from a subset of volunteers and were not included in analyses presented here.
(PDF)
