# Supplementary material for: Pseudomonas putida and Pseudomonas fluorescens Species Group Recovery from Human Homes Varies Seasonally and by Environment
Source: PLoS One. 2015 May 29;10(5):e0127704. doi: 10.1371/journal.pone.0127704 (PMC4449118; doi:10.1371/journal.pone.0127704)
Supplement: S1 Table — Upper respiratory sites were sampled in people with and without cystic fibrosis (CF); only those from people without CF were included in the analyses presented here. CF equipment sites were obtained from a subset of houses and were also not included in analyses presented here. Fecal and genital samples were obtained from a subset of volunteers and were not included in analyses presented here. (PDF) [file pone.0127704.s001.pdf]

Supporting Information.

Table S1. **Sites sampled in the homes by environment type bin.** Upper respiratory sites were sampled in people with and without cystic fibrosis (CF); only those from people without CF were included in the analyses presented here. CF equipment sites were obtained from a subset of houses and were also not included in analyses presented here. Fecal and genital samples were obtained from a subset of volunteers and were not included in analyses presented here.

Environment Type	Sampled location
Drains	Sink drain
"	Tub or shower drain
"	Floor drain
"	Garbage disposal
"	Refrigerator drain
"	Sink drain
Frequently Wet	Bath toy
"	Loofa
"	Moist or mildewed areas
"	Poof
"	Shower head
"	Soap dish
"	Spigot in tub
"	Washcloth
"	Baby bathtubs
"	Baby wipes or box
"	Bathing suit
"	Bottle drying rack
"	Bottle warmer
"	Bottles
"	Breast pump
"	Food spoons
"	Pacifier
"	Spit up cloths
"	Teething rings
"	Training toilet
"	Contact lens storage
"	Denture storage
"	Pet chew toy
"	Coffee maker
"	Dish drying rack
"	Dish-wash rag
"	Dish-wash scrubber or brush
"	Dish-wash sponge or pad
"	Base of counter backsplash
"	Ice or water; dispenser

"	Moisture in under-sink cabinet
"	Refrigerator spills
"	Water filter
"	Garden hose
"	Outdoor water toys
Soils	House plants
"	Soil around AC unit
"	Sand box
"	Soil, garden
"	Soil, yard
Surfaces	Counter
"	Tubs of cream or gel or lotion
"	Baby carrier (not car seat)
"	Baby swings
"	Car seat
"	Changing table
"	Coat
"	Crib
"	Electronic toy controls
"	Frequently worn clothing
"	High chair tray
"	Stroller
"	Toy boxes
"	Toys in crib
"	Computer keyboard
"	Pet bedding
"	Pet food bowl
"	Pet toys, (excluding chew toys)
"	Counter
"	Counter spills
"	Ground vegetables
"	Microwave
"	Refrigerator vegetable drawer
"	Stove top
"	Grill
"	Play area
Garbage and Compost	Diaper disposal container
"	Pet litter box
"	Compost or compost container
"	Garbage container (indoor)
"	Compost bins
"	Garbage cans
Water	Toilet
"	Water play table
"	Decorative fountain
"	Dehumidifier

" Fish tank
" Flower vase
" Humidifier
" Pet water bowl
" First flush of kitchen faucet
" Bird bath
" Bucket
" Fountain
" Garden ponds
" Pool or hot tub
" Standing water
" Watering can
